# Digital Health and Primary Health Care Quality: A Survey Case Study

**DOI:** 10.3390/ijerph22071015

**Published:** 2025-06-27

**Authors:** Renan Cabral de Figueirêdo, Ísis de Siqueira Silva, Pedro Bezerra Xavier, Aguinaldo José de Araújo, Amanda Jéssica Bernardo da Silva, Cícera Renata Diniz Vieira Silva, Walterlânia Silva Santos, Josemario de Abreu Silva, Severina Alice da Costa Uchôa

**Affiliations:** 1 Department of Collective Health, Federal University of Rio Grande do Norte, Natal 59078-900, RN, Brazil; isis.siqueira.176@ufrn.edu.br (Í.d.S.S.); marinhoabreusilva@gmail.com (J.d.A.S.); alicedacostauchoa@gmail.com (S.A.d.C.U.); 2 Center of Health Sciences, Federal University of Rio Grande do Norte, Nata 59012-570, RN, Brazil; pedrobx37@gmail.com; 3 Department of Dentistry, Federal University of Rio Grande do Norte, Natal 59056-000, RN, Brazil; aguinaldo.araujo@hotmail.com (A.J.d.A.); amandajessica.bernardo@gmail.com (A.J.B.d.S.); 4 Technical School of Health of Cajazeiras, Federal University of Campina Grande, Cajazeiras 58900-000, PB, Brazil; renatadiniz_enf@yahoo.com.br; 5 Nursing Department, University of Brasília, Brasilia 72220-275, DF, Brazil; walterlania@unb.br

**Keywords:** digital health, primary health care, surveys and questionnaires, health informatics, health information technology, digital health education

## Abstract

Background: Digital health, especially in Primary Health Care (PHC), has been expanding rapidly, encompassing various technologies to improve care. This study aims to evaluate the integration of digital technologies in PHC, identifying barriers and facilitators in a Brazilian capital in an urban context. Methods: A survey with a questionnaire based on a validated model was used, involving physicians and nurses from Basic Health Units. The analysis included descriptive statistics and association tests in the SPSS software, with a significance level of 5%. Results: The findings show the presence of computers and the use of e-SUS/Electronic Citizen Record in all units, highlighting WhatsApp^®^, telephone calls, and other digital media as the main used tools. It was observed that there was limited digital infrastructure, a lack of adequate training for professionals or specific protocols for the organization of digital actions and statistical associations with the performance of digital health actions. Conclusions: It is recommended to strengthen policies for professional qualification and investments in infrastructure, aiming at the continuity and improvement of the use of Information and Communication Technologies in PHC. These findings offer comparable insights for similar contexts in Brazil in urban PHC settings and countries with equivalent socioeconomic contexts and analogous public health systems.

## 1. Introduction

Information and Communication Technologies (ICTs) have been increasingly used, and their dissemination has transformed several fields, including health. In recent years, the adoption of ICTs in health services has grown remarkably, covering several areas and improving the communication and dissemination of information. This phenomenon has revolutionized the health field on a global scale, as such technologies are increasingly adopted to improve the access, efficiency, and quality of health services [1,2].

The World Health Organization (WHO) brings together the use of a wide range of technologies employed in health care, expressed in the terms telemedicine, telehealth, e-Health, mobile health (mHealth), as well as emerging areas (artificial intelligence, genomics and big data), across a broad range for the concept of digital health [3]. When analyzing the concept of digital health based on a literature review, Silva et al. [4] considered 17 terms as synonyms, showing the importance of a rapid transition in the provision of care through the scope of digital health, linking care, education, and management in Primary Health Care (PHC) as essential attributes of the concept.

The use of ICTs in remote health care strategies has had an important and rapid development, driven exponentially by the COVID-19 pandemic [2,4,5,6]. This influence can be observed in the national and international arguments issued at the time, which emphasized the applicability of ICTs in the health field. After the end of the public emergency was declared in Brazil, the professional councils encouraged the Ministry of Health to regulate the definitive use of digital health as a complementary health strategy [6].

The adoption of digital health in Primary Health Care contributes to reducing costs and travel times, achieving global development goals, improving the access, quality and longitudinality of care [7], as well as enabling more efficient regular home monitoring, increased patient satisfaction, and decreased demand for hospital care [8]. Nevertheless, the advances provided by digital health raise alerts to the challenges to be faced, such as the available technological infrastructure settings, digital health literacy, equitable access and sustainability [9,10,11].

Digital health should be an integral part of health priorities, benefit people in an ethical, safe, trustworthy, equitable and sustainable way, and should be developed with principles of transparency, accessibility, scalability, replicability, interoperability, privacy, security and confidentiality [12]. Access to ICT resources and the use of electronic tools facilitate monitoring and evaluation, producing crucial estimates as essential devices concerning the qualification of the management and organization of services, and guiding initiatives and strategies to achieve objectives and goals [13].

Digital health has the potential to minimize inequalities in access to health services; however, there are strong relationships between middle income and service accessibility challenges, where policymakers are able to change the gradients of these inequalities by implementing equitable digital services [14]. Studies have shown that populations in countries with a high concentration of income, such as Japan, the United Kingdom and the United States, faced fewer inequalities in terms of digital health [15,16,17]; in contrast, low-income nations (for example, Bangladesh and Ethiopia) face significant barriers due to limited resources and systemic gaps [14,18]. Middle-income countries, such as Brazil, India and South Africa, occupy an intermediate position, sharing common challenges related to equitable access but differing in their capacity to implement solutions due to contextual factors like governance models, health care system fragmentation, and technological infrastructure. These variations underscore that even within the same income category, tailored policies are critical to ensuring digital health equity [14,18].

In the last 10 years, Brazil has intensified the computerization process in the health field. As important strategies in this trajectory to strengthen digital health, one can mention the institution, in 2013, of the Health Information System for Primary Care (SISAB, as per its Portuguese acronym), operationalized by the e-SUS Primary Care strategy [19]; in 2015, the National Policy on Health Information and Informatics (PNIIS, as per its Portuguese acronym), employed to promote the use of ICTs to improve health work processes [20]; in 2017, the e-Health Strategy for Brazil established fundamental guidelines for digital transformation in the health sector [21]; and in 2019, the Informatiza APS Program included financial incentives to municipalities, such as support for the computerization and enhancement of PHC data [22].

Previously, in 2011, the National Telehealth Program Brazil Networks was established (Ordinance MS No. 2546/2011), demonstrating the potential of telemedicine to connect Primary Health Care (PHC) professionals with specialists by offering teleconsultations, telediagnostics, and tele-education services [23]. This program, operating through 23 state or regional centers mainly affiliated with federal universities, potentially covers the entire Brazilian PHC system [24], providing services that enhance the problem-solving capacity of PHC and consequently reduce referrals to other levels of care [24,25]. These experiences illustrate how digital technologies can strengthen the problem-solving capacity of PHC, especially in regions facing shortages of specialists and barriers to access.

In 2020, the Digital Health Strategy for Brazil 2020–2028 (ESD28, as per its Portuguese acronym) was implemented, embodied in the strategic vision of updating and expanding digital health and in its action, monitoring and evaluation plan [26], and the National Health Data Network, a national platform for the integration of health data between different systems and care units [27]. In 2023, the Information and Digital Department of Health (SEIDIGI, as per its Portuguese acronym) was founded to formulate public policies aimed to promote innovation and adopt emerging technologies in the health sector [28]. Finally, in 2024, there was the Institution of the SUS Digital Program, with the purpose of boosting a digital transformation within the scope of the Brazilian Unified Health System (SUS, as per its Portuguese acronym) [29].

Thus, it is essential to understand the various applications of digital health today, investigating and exploring how different actors interact with them and in which specific contexts these resources can be optimized, taking into account the characteristics of each medium, platform and culture [30]. The challenges of digital technology implementation in public services extend beyond health care, with similar patterns observed in e-government initiatives and digital inclusion programs [31,32]. Research on digital divide and technology adoption reveals that the Brazilian experience with digital health reflects broader patterns of unequal technology access and implementation challenges faced across multiple sectors [33]. In this sense, it is recommended that evaluative studies be carried out to contemplate the local realities of this PHC practice in the various settings, unveiling its impacts on the quality of care and its innovative care approaches [4].

Some evaluative studies already carried out have raised important questions about digital health. O’brien et al. [34] and Lennon et al. [35] used qualitative approaches to explore strengths and weaknesses, in addition to examining barriers and facilitators for the implementation of digital health, both those from the perspective of the multidisciplinary experiences of PHC professionals and those through the evaluation of a national digital health program. In Brazil, Sarti and Almeida [24], based on secondary data, analyzed the use of a telehealth program within the scope of PHC, identifying the prevalence of telehealth use and related factors. Nichiata and Passaro [36] mapped and analyzed digital initiatives in the SUS, such as health applications for mobile devices, to support communication and digital health policies. Mélo et al. [37] identified challenges and potentialities relevant for implementing and using e-SUS in Primary Health Care. Rachid et al. [38] outlined the path of public health informatics policies in the country and pointed out criticisms of the process, associating digital health with a status of “platformization” of the Brazilian state.

Digital health, when incorporated into PHC, the preferred gateway and basis of health systems, plays a crucial role in strengthening public health and meeting the Sustainable Development Goals [39]. The improvement of a universal system is deeply related to the quality of access to health care, and digital health offers valuable contributions to the structuring of health services and initiatives in PHC and in the Brazilian Unified Health System (SUS), taking into account the vast size of Brazil and the remote communities with scarce resources for access to health professionals and services [40].

Although there are studies on digital health in Brazilian PHC, a critical gap persists in that most research is not based on systemic models for evaluating health quality, such as Donabedian’s model [41], the essential attributes of PHC [42], and the WHO’s quality of care elements [43]. This theoretical disconnect limits the transformation of evidence into practical improvements, highlighting the need for research that combines these frameworks to guide continuous cycles of improvement in digital health services.

Given the dearth of studies that systematically evaluate the implementation of digital health in PHC, particularly those grounded in systemic quality frameworks, the current study aims to evaluate the integration of digital technologies in PHC, identifying barriers and facilitators in a Brazilian capital in the setting at stake.

## 2. Materials and Methods

### 2.1. Study Design

This is a quantitative survey-type study that collects data on the attributes, actions or perceptions of a specific group of individuals, representative of a larger population, using a research instrument, usually a questionnaire, for a given topic [44]. The survey approach was chosen for its ability to capture perceptions and experiences in a systematic and quantifiable way, allowing for the comparison of findings, the identification of patterns, barriers and facilitators, and providing robust evidence for public policies as well as improvements in terms of implementing technologies.

### 2.2. Context

The research was developed in 2024, with collection in virtual and on-site environments, in the context of the PCH network of Natal/Rio Grande do Norte (RN), Brazil. According to the regionalization master plan, RN is divided into eight Regional Health Units (URSAP, as per its Portuguese acronym), with Natal being the headquarters of the VII URSAP, which covers four other municipalities and concentrates the largest population in the state, standing out as an important economic and cultural center [45].

The choice of Natal as the place of this study is justified by its relevance within the state of RN and in the context of health regionalization, in addition to its strategic location and emerging technological infrastructure. As the most populous city in the state, with 751,300 inhabitants [46], Natal faces challenges typical of metropolitan regions, such as the need to optimize resources and improve the efficiency of health services. With an area of 167.4 km^2^ and a demographic density of 4488.0330 [46] inhabitants per km^2^, the city has characteristics that allow for studying how a medium-sized capital can integrate digital solutions into PHC. These insights may be applicable to other cities with similar characteristics or may even serve as a model for smaller municipalities or those in the early stages of health computerization. In terms of connectivity, the city has 236.8 thousand fixed broadband subscriptions and 1.1 million mobile phone accesses, with more than 90% having mobile internet access [47].

The capital joined the Informatiza APS Program [22] and has a total of 58 Basic Health Units (BHUs), distributed in 5 health districts. They are the North I health district, with 12 BHU; the North II district, also with 12 BHU; the South district, with 10 BHU; the East district, with 8 BHU; and the West district, with another 16 BHU [48]. In June 2024, based on the National Registry of Health Establishments, Natal had 147 active Family Health teams (eSF, as per its Portuguese acronym) and 42 Primary Care teams (eAP, as per its Portuguese acronym) [49].

### 2.3. Population, Sampling and Sample

The study population was composed of medical professionals and nurses from the eSF and eAP teams in the city of Natal/RN, whose most recent consultation showed a total of 357 registered professionals, 176 physicians and 181 nurses [49]. A stratified probabilistic sampling process was carried out [50], considering the BHU facilities as units of stratification and representation of the sample subjects in all establishments, as well as in the 5 health districts of the capital under study.

All professionals targeted by the study (357) were included, while those who met the following criteria were excluded: professionals on leave for various reasons (vacations, illnesses, leaves, among others), those who retired during the collection period, those who refused to participate in the study, and those who did not answer the form after at least three requests. As a result, the final sample was composed of 256 respondents, which corresponds to 71.71% of the study population.

### 2.4. Instrument and Data Collection

The data collection instrument used was “Instrument 1—Questionnaire/Form for PHC Physicians and Nurses,” based on the “QualiAPS Digital—Brazil” indicator matrix. This tool was developed, validated, and adapted to the Brazilian Primary Health Care (PHC) context in a previous study conducted by the authors Figueirêdo et al. [40]. The matrix was structured according to Donabedian’s classic health evaluation triad (structure, processes, and outcomes) [41], while also incorporating WHO recommended dimensions of health care quality (efficacy, efficiency, safety, timeliness, integration, equity, and people-centered care) [43] and Starfield’s essential PHC attributes: first-contact access, longitudinality, comprehensiveness, and coordination [42]. This framework enables a systematic analysis of how digital technologies influence service organization and integration.

The instrument used contains 40 questions, 35 of which are multiple-choice and 5 which are open, as shown in the Supplementary Materials; it can be accessed in the supplementary material 3 (pp. 1–7) in Figueirêdo et al. [40]. A brief description of the instrument can be found in Table 1 below:

In order to start the fieldwork, a previous contact was made with the technical team of the Municipal Department of Health via e-mail and a workshop was held in such a way as to establish the partnership, explain the research and show the commitment to research ethics, the objectives and the applicability of the study, with a view to ensuring adherence and expanding the participation of the study population.

Data collection took place between February and July 2024, initially through an electronic form (Google Forms^®^) that was sent to the entire study population attached to the official memorandum of the capital, conveyed by e-mail and messaging application (WhatsApp^®^). In order to minimize loss of responses, on-site visits were made to all BHU facilities to reinforce the collection. During these visits, initial contact was sought with the managers of the units in order to reach the professionals who were the targets of this research. They were offered the option of answering the printed form at the time, at a later and more convenient time, or even remotely, via the online form already available. Repeated contacts were made via telephone, e-mail and messaging applications until the final sample of the study was reached, generating the database available in the “Data Availability Statement” section of this article.

### 2.5. Variables and Data Analysis

The independent variables adopted were health district, type of health unit; type of employment link; age group; training and graduate training. The dependent variables were course/training for the use of digital health tools; computer equipment; response time of computer equipment; functionality of computer equipment; usability of computer equipment; internet availability for professionals; unit’s internet quality; response time of information system; functionality of information system; usability of information system; implementation of a project or set of digital health actions; temporality of digital health actions; suitability of the unit’s physical infrastructure; suitability of the unit’s technological infrastructure; ICT tools used; suitability of information system for remote service; existence of protocols, guidelines and regulations for remote care; digital health actions in the COVID-19 pandemic; target audience of digital health actions in the pandemic; continuity of post-pandemic actions; post-pandemic digital health actions; target audience of post-pandemic digital health actions; relevance of actions; suitability of digital health tools; and digital health resolvability.

The analysis specifically addressed two principal hypotheses grounded in established theoretical frameworks for technology adoption and digital health implementation:

**Hypothesis** **1** **(H1).** Better digital infrastructure conditions would be associated with the implementation of digital health actions. This hypothesis is theoretically grounded in the Technology Acceptance Model (TAM) and Donabedian’s structure-process-outcome framework [41,51]. According to TAM, perceived usefulness and ease of use are fundamental determinants of technology adoption, both of which are directly influenced by infrastructure quality [51,52]. In the context of PHC, studies have consistently shown that adequate technological infrastructure, including reliable equipment, stable internet connectivity, and clear operational protocols, constitutes a prerequisite for successful digital health implementation [53,54]. Kosowicz et al. [55] demonstrated that the absence of standardized guidelines compromises safety and uniformity in digital practices, while Nakayama et al. [10] identified unsuitable infrastructure as a primary barrier to telehealth adoption in middle-income countries. Furthermore, Donabedian’s model posits that structural elements (resources, protocols, equipment) directly influence process quality, making infrastructure adequacy a critical predictor of digital health practice implementation [41]. We evaluated this hypothesis through equipment adequacy, physical infrastructure suitability, and the presence of protocols as structural determinants of digital health action implementation.

**Hypothesis** **2** **(H2).** Professionals with digital health training would be more likely to implement digital health actions. This hypothesis is supported by adult learning theory, the diffusion of innovations model, and extensive evidence on the role of professional competency in technology adoption [56,57]. Rogers’ diffusion of innovations theory identifies knowledge and skills as essential prerequisites for innovation adoption, with training serving as a critical mechanism for building both technical competence and confidence in new technologies [56]. In health care settings, systematic reviews have demonstrated that formal training programs significantly increase the likelihood of digital tool adoption and improve implementation outcomes [58,59]. Aisyah et al. [60] specifically emphasized that continuous training is essential for the effective integration of digital technologies into clinical practice, while Eccles et al. [61] showed that structured digital health education enhances both adoption rates and the quality of implementation. The theoretical foundation is further supported by Bandura’s social cognitive theory, which posits that self-efficacy—developed through training and guided experience—is a primary determinant of behavioral change and technology adoption [62]. We evaluated this hypothesis by comparing digital health implementation rates between professionals who received and those who did not receive specific digital health training across different time periods (pre-pandemic, pandemic, and post-pandemic).

These hypotheses are particularly relevant in the Brazilian PHC context, where digital health policies have been rapidly implemented without necessarily ensuring adequate infrastructure or training preparation [26,28]. Understanding the relative importance of structural versus educational factors can inform more effective implementation strategies and resource allocation decisions for sustainable digital health integration in similar middle-income country contexts.

The statistical analysis performed in this study was based on a variety of statistical methods, including descriptive measurements and hypothesis testing. These analyses specifically examined the following: (a) associations between infrastructure variables (equipment adequacy, unit infrastructure, protocols) and implementation outcomes; and (b) differences in the implementation of digital action between trained and untrained professionals across different time periods. The adopted descriptive measurements were absolute and percentage frequencies, describing the characteristics of the variables and providing summarized information about the collected data. The Chi-square test was used to investigate the association between different categorical variables. This test allowed us to evaluate whether the observed frequencies differed from the expected frequencies, indicating possible statistically significant associations between the variables [63]. Fisher’s exact test was applied when the sample size was small, thus allowing us to evaluate the association between two categorical variables when the conditions of applicability of the Chi-square test were not met [64]. In the current study, statistical analyses were performed using the Statistical Package for the Social Sciences (SPSS) software (version 25.0) and the adopted significance level of 5%.

### 2.6. Ethical Aspects

The study in question was approved by the Research Ethics Committee of the Onofre Lopes University Hospital of the Federal University of Rio Grande do Norte (CEP HUOL/UFRN) under CAAE nº 48655521.9.0000.5292 and Opinion nº 4.859.682.

## 3. Results

The descriptive data on the characteristics of the responding professionals, as well as the available infrastructure and the professionals’ perceptions about the used digital tools and information systems, are displayed in Table 2. Most of the professionals are distributed in the West (30.47%), North II (18.36%) and North I (17.19%) health districts. Regarding the type of unit, most of them work in Family Health Units (FHUs), representing 73.05% of the respondents, while 21.88% are in Basic Health Units (BHUs). With regard to employment, more than half of the professionals are statutory (50.39%), followed by hired (23.83%) and those linked to the More Physicians Program (17.19%). The predominant age group is between 31 and 40 years old (39.84%), followed by the group aged from 41 to 50 years old (20.31%). From the educational aspect, the sample is made up of nurses (53.91%) and a considerable portion of physicians (46.09%), with 74.61% of professionals having a graduate degree. In terms of training, 78.91% of professionals did not take specific courses or training for the use of digital health tools.

Although all units have computers (100%) and the vast majority printers (91.80%), the availability of other computer equipment, such as laptops (8.59%) and tablets (0.39%), is limited. The respondents expressed criticisms regarding the quality of such equipment (crashes in 79.20% and response time of moderate to slow, 60.55% and 26.17%, respectively). In addition, the quality of the internet connection varied, with 45.42% of respondents classifying it as good and 34.58% considering it average. The information system used was the e-SUS/PEC, but there are criticisms regarding the performance of this system, with 67.19% of respondents stating that the response time is moderate and 88.58% reporting frequent crashes. Regarding usability, 85.55% of professionals consider the systems easy to use.

When the research participants were asked about the implementation of projects or sets of digital health actions in their health teams, there was a prevalence of negative answers (72.68%). Most of the health actions through digital media (66.67%) occurred during the period of social distancing and the temporary closure of health services during the COVID-19 pandemic and consisted mostly of guidance and remote care of COVID-19 cases (62.16%), with a greater concentration on adults (88.24%) and elderly people (76.47%). After the critical period of the pandemic (from the second half of 2022), only 25.77% of professionals reported that they continued to develop digital health actions, this time with greater emphasis on disease prevention (70.59%) and health promotion (76.47%) actions, with adults (92.11%) and women (80.26%) standing out. In terms of the suitability of the informatics infrastructure, 76.57% of professionals reported that the equipment was not suitable to deal with on-site and remote demands. Similarly, 83.08% indicated that there was no suitability of the physical infrastructure of the health units for this transition.

As for the ICT tools used in digital actions, one can highlight the use of the WhatsApp^®^ messaging application (65.45%), telephone calls (60%), and the Instagram social network (50%). A considerable portion of the professionals judge the used information system (e-SUS/PeC) as unsuitable for remote care (62.79%) and state that there is a lack of protocols for the organization of these actions (84.78%). Regarding the problems faced by them, the professionals recognized the relevance of remote care actions most of the time (40.57%) and sometimes (30.19%), as well as the suitable digital tools for this most of the time (43.70%) and sometimes (29.41%), with a resolvability rate for digital health of around 78.57%. These data can be seen in Table 3.

Table 4 displays the analysis of the professionals’ characteristics in relation to the performance of digital health actions, with a comparison between the groups that do not perform (*n* = 188) and those that perform (*n* = 68) these actions, using the Chi-square test to evaluate associations. The age groups of professionals are divided between those over and under 40, with 44% of the total being over the age of 40; further, 41% among those who do not perform digital actions and 51% among those who perform these actions and are over 40 years old, without significant association (*p* = 0.155). The age group from 20 to 40 years old comprises 56% of the total, having a greater presence in the group that does not perform digital actions (59%) compared to those who perform them (49%). As for the employment link, 50% of professionals have a statutory relationship, with 52% among those who do not perform and 46% among those who perform digital actions. The other employment links represent 50% of the total, without significant difference between the groups (*p* = 0.355).

Regarding professional training, 54% are nurses, with a higher proportion among those who perform digital actions (60%) compared to those who do not perform them (52%). Physicians correspond to 46%, distributed among the groups (*p* = 0.218). Concerning graduate studies, 75% of professionals have this training, with a higher proportion among those who perform digital actions (81%) than among those who do not perform them (72%), without significant association (*p* = 0.165). When considering training for the use of digital tools, 79% of professionals did not receive specific training, with most of them in the group that does not perform digital actions (81%). About 26% of the group that performs digital actions are among those who have taken courses, against 19% for those who do not perform digital actions (*p* = 0.205). These findings regarding training showed no significant associations with the implementation of digital health actions. As for suitability, the unit’s computer equipment was mentioned by 77% as unsuitable, with greater unsuitability in the group that does not perform digital actions (82%) compared to those that perform them (64%), showing a significant association (*p* = 0.013).

Supporting our infrastructure hypothesis, the unit’s physical infrastructure was considered unsuitable by 83% of professionals, with 89% in the group that does not perform digital actions and 69% in the group that performs these actions, with a significant association (*p* < 0.001). Further supporting our infrastructure hypothesis, the presence of protocols and guidelines for remote care actions is low (15%), with 91% of professionals in the group that does not perform digital actions reporting the absence of these regulations, while 68% of the group that performs them also stated the absence, showing a significant association (*p* < 0.001). Finally, digital health resolvability was evaluated as positive by 79% of professionals, without significant difference between the groups (*p* = 0.645). These data highlight the influence of infrastructure and regulatory aspects on the practice of digital health actions, with significant associations for the suitability of equipment and infrastructure, in addition to the presence of guidelines.

Table 5 compares health professionals who took a course or training for the use of digital health tools with those who did not perform them, using Fisher’s exact test and Chi-square test for independence to evaluate differences between the two groups. Partially supporting our training hypothesis (H2), as for the temporality of digital health actions before the pandemic, professionals who underwent training for the use of digital tools were significantly more likely to implement these actions (43%) compared to those who did not undergo training (11%), with a *p*-value = 0.009, indicating a statistically significant difference. During the period of social distancing, 75% of untrained professionals performed digital actions, while 52% of those trained also did so, although this difference was not significant (*p*-value = 0.081). This pandemic-period finding does not support H2 expectations.

After the second half of 2022, the proportions of continuity of digital actions were similar between the two groups, with a *p*-value = 0.518. Providing nuanced support for H2, in the digital actions performed during the critical period of the pandemic, telecare for cases unrelated to COVID-19 was significantly more frequent among professionals who underwent training (17%) than among those who did not undergo training (2.5%), with a *p*-value = 0.015. Nonetheless, for the other actions, such as guidance and remote care of COVID-19 cases, telecare for COVID-19, continuity of treatment for Chronic Non-Communicable Diseases (NCDs), and prevention and health promotion actions, there were no significant differences between the groups. Contrary to H2, disease prevention actions performed today were more frequent among professionals who did not undergo training (78%) than among those who underwent training (50%), with a *p*-value = 0.025, indicating a statistically significant difference. For other actions, such as the continuity of NCD treatment and health promotion, there were no significant differences. Regarding resolvability, no statistically significant differences were observed between the opinions of the groups that had or had not taken a course/training for the use of digital health tools. Overall, these mixed results offer partial but inconclusive support for H2 regarding training effects.

Table 6 compares the characteristics of nurses and physicians who developed digital health actions during the COVID-19 pandemic and its continuity. Regarding the temporality of digital health actions before the pandemic, the proportions of nurses (23%) and physicians (24%) who implemented digital practices were similar, without significant difference (*p*-value > 0.999). During the period of social distancing during the pandemic, 73% of nurses stated that they developed digital actions, compared to 53% of physicians, a difference that was not statistically significant (*p*-value = 0.152).

After the second half of 2022, the proportions were 20% of nurses and 29% of physicians (*p*-value = 0.499). Regarding digital actions, during the critical period of the pandemic, nurses (68%) and physicians (51%) reported providing remote guidance and care for COVID-19 cases, with a tendency towards statistical significance (*p*-value = 0.097). In other actions, such as telecare for COVID-19, continuity of treatment for Chronic Non-Communicable Diseases (NCDs), prevention actions and health promotion, there were no statistically significant differences between the groups. The continuity of digital health actions also did not show significant differences between nurses and physicians, except for a trend in the continuity of NCD treatment, which was more frequent among physicians (52%) than among nurses (29%), with *p*-value = 0.061. Although the adequacy of IT equipment to meet remote demands was slightly higher for nurses (27%) than for physicians (19%), this difference was not statistically significant (*p*-value = 0.240). As for physical infrastructure, there was a trend towards significance, with physicians reporting lower adequacy (11% versus 21%, *p*-value = 0.051).

## 4. Discussion

The data found on the characteristics of the health professionals indicate a predominance of nurses who were available to answer the questionnaire and a high proportion of professionals with graduate training. This profile, in theory, could favor the adoption of digital practices since qualification and experience could potentially facilitate adaptation to new technological tools. Nevertheless, the lack of specific training in ICTs, as reported by most respondents, suggests that academic training has not been accompanied by training aimed at digital health. Authors like Aisyah et al. [60] et al. highlight the need for continuous training to effectively integrate digital technologies into clinical practice. The absence of training limits the appropriate use of digital tools, affecting the efficiency and quality of practices.

Our findings strongly confirm Hypothesis 1 (H1) regarding infrastructure limitations. The analysis of the technological infrastructure of the health units surveyed exposes significant challenges. Although all units in Natal have computers and most have printers, the shortage of equipment, such as tablets and laptops, restricts the flexibility of digital health practices. The low quality of computer equipment evidenced by the results, with frequent crashes and slowness, corroborates the barriers identified by Nakayama et al. [10], which highlight the importance of a stable and robust technological environment for effective digital practices in PHC. Insufficient connectivity, reported by many respondents as only average, limits the use of digital practices that require a stable connection, preferably broadband, compromising the potential of interactive and integrated tools.

The data on digital health care actions indicate that digital practices were especially intensified during the critical period of the COVID-19 pandemic, with a predominant use of WhatsApp^®^ and telephone calls. This adaptive use of digital tools, driven by the need for social distancing, is well documented in Silva et al. [2], showing the increased use of ICTs as a response to the crisis. The discontinuity of these digital practices after the pandemic period, reported by more than 70% of respondents in this study, emphasizes a lack of institutionalization of these actions. Without support in sustainable policies and resources, this temporary dependence limits the lasting impact of digital practices in PHC, as Araújo et al. warn [6]. It is believed that this decline is attributable to the return of on-site activities, with an increase in other demands and a lack of local policies to promote digital health care, such as adequate professional training, investments in physical and technological infrastructure, and the adoption of protocols for activities.

The incorporation of new concepts and recent sociotechnical advances, such as social media applications, has made the notion of digital health more comprehensive [65], and this use as a means of disseminating health information can be questioned, especially in terms of its safety. Nevertheless, tools like Instagram^®^ and WhatsApp^®^ have been increasingly used to facilitate and speed up the dissemination and exchange of information, as well as for clinical decision making, promotion of health education, support for patients during treatment, and even communication between professionals and the care network [66,67,68,69,70]. Official institutional accounts and profiles can help to increase the reliability of these channels.

The infrastructure challenges central to H1 are further exemplified with regard to the adequacy of digital tools and the information system, and the data denote a digital infrastructure characterized mainly by the presence of computers, wired internet, and the training system/Citizen’s Electronic Medical Record (e-SUS/PEC, as per its Portuguese acronym). Many professionals consider the e-SUS/PEC system insufficient for remote care due to technical and operational limitations, such as predominantly moderate response time (67.19%) and frequent crashes (88.58%). This problem is common in contexts with limited infrastructure, as observed by Ladaga et al. [66], who highlight that tools without appropriate support lose effectiveness for remote care. The lack of specific protocols to organize these digital actions represents an additional barrier, according to Kosowicz et al. [55], who indicate that the absence of standardized guidelines compromises safety and uniformity in digital practices.

The statistical associations supporting H1 emerge clearly when comparing groups that perform and those that do not perform digital actions, suggesting a direct influence of structural conditions on the digital health practice. The suitability of equipment and physical infrastructure is significant for the implementation of digital actions, with statistical associations indicating that the low quality of infrastructure and the lack of guidelines directly influence the ability of professionals to conduct digital practices. These findings reinforce the importance of suitable infrastructure for the use of ICTs, especially in low- and middle-income countries, as discussed by Delponte et al. [71] and Omotosho et al. [72], who point to unsuitable infrastructure as one of the main barriers to success in terms of digital health.

These infrastructure challenges are likely magnified in rural settings, where connectivity issues and limited technological resources could create additional barriers for infectious disease prevention and management, particularly for implementing remote monitoring and quarantine protocols that became critical during health emergencies [70].

Another relevant point displayed is the impact of the training of professionals on adherence to digital practices. Professionals with specific training in ICTs showed a greater propensity to perform teleservices, especially for conditions unrelated to COVID-19 during the pandemic, highlighting the importance of continuous and specific training. Interestingly, while H1 focused on infrastructure, the higher frequency of preventive actions among those without training suggests a creative adaptation of professionals in the context of limitations. Eccles et al. [61] point out that training is essential, but that practical experience can also drive the adoption of digital practices, even in the face of limited resources.

The practical challenges identified in our study align closely with findings from Lennon et al. [35], who emphasized that digital health implementation requires addressing both technical and organizational barriers at the frontline level. Our field evidence reveals specific operational difficulties that illustrate these broader challenges. For instance, the frequent system crashes reported by 88.58% of professionals using e-SUS/PEC lead to daily workflow disruptions that force health care workers to develop informal workarounds, such as reverting to paper records or using personal devices for patient communication. The moderate-to-slow response times experienced by 86.36% of users (60.55% moderate + 26.17% slow) result in bottlenecks during patient consultations, with professionals reporting frustration when electronic systems delay rather than facilitate care delivery. These technical limitations are compounded by the absence of adequate training, as evidenced by professionals who learn to use the system through trial and error, often discovering functions by accident rather than through formal instruction.

The infrastructure inadequacy extends beyond equipment to spatial constraints, with 83.08% reporting unsuitable physical infrastructure—a challenge that became particularly evident during the pandemic when professionals needed to conduct teleconsultations in shared spaces without privacy or adequate internet connectivity. As noted by Lennon et al. [35], successful digital health implementation requires sustained organizational support and iterative adaptation based on user feedback. Our findings suggest that while Brazilian PHC professionals demonstrate remarkable adaptability—evidenced by the creative use of WhatsApp^®^ and informal communication channels—this adaptation occurs not because of, but in spite of, systematic institutional support. This pattern mirrors international experiences where frontline innovation often compensates for inadequate digital health infrastructure, but such solutions remain fragile without formal integration into health system processes [34,35].

Finally, health professionals’ evaluation of PHC’s problem-solving capacity through digital health tools was predominantly positive (78.57%), indicating that these technologies are perceived as effective for addressing primary care demands without requiring referrals. This finding aligns with the Starfield [42] first-contact principle, reinforcing digital health’s potential to enhance resolvability in PHC, provided it is supported by adequate infrastructure and clear protocols, being able to produce a positive impact on users’ quality of life, focusing on attention to their health needs, and aiming for a comprehensive approach, effective responses and relief or minimization of suffering [73].

The H2 perspective reminds us that the literature supports the integration between ICTs and appropriate training, as it can improve the efficiency and effectiveness of care in PHC. Reis [74] reinforces that the structured use of digital health strengthens the bond with patients and optimizes the time of care. Nevertheless, for this problem-solving capacity to be sustained, it is essential that policies guarantee infrastructure and continuing education, ensuring the positive and lasting impact of digital practices.

Our findings align with broader technology adoption literature from sociology and information systems fields, which emphasizes that successful digital transformation requires not only technical infrastructure but also social and organizational readiness [75]. The pragmatic adaptation observed among PHC professionals—using WhatsApp^®^ and informal communication channels—reflects patterns documented in innovation studies, where users creatively appropriate technologies to meet their needs despite inadequate institutional support [76].

An important aspect that emerges from the implementation of digital health practices in PHC is the need to balance the protection of privacy with the necessary sharing of medical information for effective care. In Brazil, this tension is governed by the General Data Protection Law (Lei Geral de Proteção de Dados—LGPD), enacted in 2018, which establishes strict guidelines for the processing of personal data, including health information [77]. The LGPD recognizes that health data require special protection while allowing their sharing when justified by legitimate public health interests or vital patient needs [78]. In emergency situations, the law permits the sharing of data without explicit consent when necessary to protect the patient’s life or physical integrity, thus creating a legal framework that balances individual privacy with collective health needs [77,78]. However, the implementation of digital health tools in PHC raises practical challenges related to obtaining informed digital consent, especially in cases where informal platforms such as WhatsApp^®^ are employed for health communication. The lack of specific protocols for digital consent and data security in remote care, reported by 84.78% of professionals in our study, highlights the urgent need for clear guidelines that ensure both privacy compliance and effective health care delivery. This regulatory gap represents a significant barrier to the systematic adoption of digital health practices and underscores the importance of developing comprehensive protocols that address both technical security requirements and ethical considerations in digital health implementation.

### Study Limitations and Potentials

This study was conducted in one capital (Natal/RN), which may limit the generalizability of the results to other contexts. However, the methodological framework, grounded in Donabedian’s structure–process–outcome model and Starfield’s PHC attributes, provides a theoretical foundation that enhances transferability to similar urban settings in middle-income countries. While the sample was not calculated a priori, the inclusion of 100% of health units and 71.71% of eligible professionals ensures representativeness for the local context. The focus on frontline workers offers a critical view of structural and operational barriers; even so, the inclusion of the perspective of managers and users is recommended for future studies. These limitations are counterbalanced by the study’s rigorous application of a validated instrument named, “QualiAPS Digital—Brazil”, which aligns with global frameworks for digital health evaluations and supports comparative analyses.

Another important limitation of this study is its exclusive focus on an urban setting (Natal/RN), which may not capture the digital health challenges faced by rural and remote communities. Urban areas typically have better technological infrastructure, internet connectivity, and proximity to specialized services compared to rural regions [10]. In rural contexts, digital health tools may play an even more critical role in infectious disease prevention and management, given the limited access to health care facilities and the challenges of implementing quarantine and isolation measures in geographically dispersed populations [79,80]. Rural communities often face unique barriers including limited broadband access, lower digital literacy rates, and distinct social dynamics that could significantly impact the adoption and effectiveness of digital health interventions [81]. Future research should specifically examine digital health implementation in rural PHC settings to understand how geographic context influences the integration of digital technologies, particularly for infectious disease surveillance, contact tracing, and remote monitoring capabilities that were proven to be essential during the COVID-19 pandemic [79,82].

## 5. Conclusions

This study presents the first in-depth diagnosis of digital health in Primary Health Care (PHC) in Natal/RN, based on an integrative theoretical framework that articulates Donabedian’s structure–process–result model with the fundamental attributes of PHC defined by Starfield, such as universal access, longitudinality, comprehensiveness and coordination of care. This approach, widely recognized in the evaluation of PHC, is applied here in an innovative way to the field of digital health, making it possible to understand how technological infrastructure directly impacts the capacity of services to provide solutions. In addition to highlighting the existing structural and operational challenges, the study proposes a replicable evaluation model to measure digital integration in PHC, offering concrete subsidies for evidence-based public policies, with the potential to improve the quality of care, health outcomes and equity in access.

In Natal, the results show that, despite the wide availability of basic equipment (100% of units have computers), the digital infrastructure has significant weaknesses (79.2% of professionals reported frequent equipment failures and 76.57% considered the structure inadequate for on-site and remote demands). These technical limitations are aggravated by organizational shortages, with 84.8% of units lacking protocols for remote care and 83.08% not physically adapted to the new digital model of care. However, even in this challenging scenario, the professionals’ perception of the resolution potential of digital tools was mostly positive (78.57%), indicating fertile ground for structured interventions.

The analysis also revealed that professional training emerges as a crucial factor for technological adoption, as evidenced by the strong association between the training itself and the effective use of the tools (*p* < 0.05). However, 81% of professionals had not received adequate training, highlighting a disconnect between technological implementation and the preparation of human resources. This finding gains particular relevance when one observes that the most used tools are informal platforms like WhatsApp^®^ (65.45%) and telephone calls (60%), suggesting a pragmatic but unsystematized appropriation of the available technologies.

The contributions of this study can be seen on three complementary levels. On the theoretical–methodological level, one can progress in proposing the application of an integrated framework that overcomes the methodological fragmentation of the national literature, where most studies are restricted to isolated technological evaluations. In terms of practical management, it offers a pioneering operational diagnosis, not only in Natal but which can be inferred for other cities, given that the national digital SUS strategy has not been undertaken. The study identifies clear priorities: (1) investment in stable infrastructure; (2) development of protocols for remote care; and (3) ongoing training programs. As a tool for public policy, the study provides adaptable indicators for monitoring the SUS Digital strategy in different local contexts.

By highlighting significant structural and operational limitations, the study demonstrates that, despite the urban focus, these challenges reflect issues shared by many middle-income municipalities. However, the urban context may not fully represent the additional complexities faced by rural communities, particularly regarding infectious disease prevention and management, where digital health tools could be even more crucial due to geographic isolation and limited health care access. This limitation raises important questions about digital equity and the need for tailored approaches that consider the specific challenges faced by rural PHC settings.

## Figures and Tables

**Table 1:** Distribution of the questions contained in the instrument by theme, component, dimension and indicator in the “QualiAPS Digital—Brazil”.

Sample Characterization			
Questions 1 to 10	Variables: type and name of the health unit, professional affiliation with the municipal health department, age, education, year of completion, whether the person has a graduate degree and which one, whether the person has a course/training for the use of digital tools in health and which one.		
Theme 1: Digital Infrastructure in Health Care Facilities			
Questions	Indicators *	Dimensions	Components
1 to 5	R8. Access to and quality of equipment and tools for operationalizing digital health in Primary Health Care (PHC).	Resources (R) (10 questions)	Structure
6 to 7	R9. Internet availability and quality in the Health Unit.		
8 to 10	R11. System quality (e-SUS/ECR or municipal management’s own systems).		
Theme 2: Team Work Processes			
11 to 12	R2. Number of Primary Health Care/Family Health Unit teams that have implemented a digital health project, program or set of actions.	Resources (R) (11 questions)	Structure
19 to 20	R7. Adequacy of the physical and technological infrastructure of health units to meet multiple demands		
21	R2. Number of managers who have implemented a project, program or set of digital health actions in PHC.		
22	R5. Categories of professionals involved in remote care at the unit, district or central level.		
23	R3. Number of Family Health/Primary Health Care teams that use/used remote care in PHC		
24	R.10 Digital tools used in remote care		
25	R11. System quality (e-SUS/ECR or municipal management’s own systems).		
26 to 27	R14. Existence and adequacy of protocols, guidelines and regulations for the organization of digital health actions.		
13 to 18	T1. Digital health actions carried out by Family Health/Primary Health Care teams in remote care	Technique (T) (6 questions)	Process
28 to 30	MT1. Effectiveness (relevance and appropriateness of technology choices in relation to health problems) and resoluteness of digital health.	Medium Term Results (MT) (3 questions)	Results

* The abbreviation of the indicators by letters and numbers corresponds to their identification in the “*QualiAPS Digital—Brazil*” matrix (Supplementary Material—Data Sheet 5) [40].

**Table 2:** Characteristics of health professionals, technological infrastructure of health units and the professionals’ perception about the digital tools used.

Characteristics	*n*/*N* (%)
Sanitary district, *n*/*N* (%)	
East	40/256 (15.63%)
North I	44/256 (17.19%)
North II	47/256 (18.36%)
West	78/256 (30.47%)
East	47/256 (18.36%)
Type of health unit, *n*/*N* (%)	
BHU	56/256 (21.88%)
BHU/FHU	13/256 (5.08%)
FHU	187/256 (73.05%)
Employment link, *n*/*N* (%)	
Hired	61/256 (23.83%)
Cooperative	10/256 (3.91%)
Statutory	129/256 (50.39%)
More Physicians Program	44/256 (17.19%)
Physicians for Brazil Program	10/256 (3.91%)
SESAP	1/256 (0.39%)
Outsourced	1/256 (0.39%)
Age group, *n*/*N* (%)	
+60	26/256 (10.16%)
20–30	41/256 (16.02%)
31–40	102/256 (39.84%)
41–50	52/256 (20.31%)
51–60	35/256 (13.67%)
Training, *n*/*N* (%)	
Nurse	138/256 (53.91%)
Physician	118/256 (46.09%)
Graduate training, *n*/*N* (%)	
No	65/256 (25.39%)
Yes	191/256 (74.61%)
Have a course or training in the use of digital health tools, *n*/*N* (%)	
No	202/256 (78.91%)
Yes	54/256 (21.09%)
Computer equipment of the health unit, *n*/*N* (%)	
Computers	256/256 (100.00%)
Laptops	22/256 (8.59%)
Tablets	1/256 (0.39%)
Wireless routers	34/256 (13.28%)
External hard drives	2/256 (0.78%)
Network servers	84/256 (32.81%)
Routers	86/256 (33.59%)
Printers	235/256 (91.80%)
Webcams	1/256 (0.39%)
Microphones	20/256 (7.81%)
Speakers	66/256 (25.78%)
Response time of computer equipment, *n*/*N* (%)	
Slow	67/256 (26.17%)
Moderate	155/256 (60.55%)
Fast	34/256 (13.28%)
There are crashes, *n*/*N* (%)	
No	52/250 (20.80%)
Yes	198/250 (79.20%)
Easy to use, *n*/*N* (%)	
No	24/254 (9.45%)
Yes	230/254 (90.55%)
Information system used in the health unit, *n*/*N* (%)	
There is no information in the unit	1/256 (0.39%)
It uses the e-SUS/PEC—APS system	255/256 (99.61%)
Internet availability for professionals, *n*/*N* (%)	
No	96/251 (38.25%)
Yes	155/251 (61.75%)
Perception of internet quality, *n*/*N* (%)	
Excellent	13/240 (5.42%)
Good	109/240 (45.42%)
Average	83/240 (34.58%)
Bad	29/240 (12.08%)
Awful	6/240 (2.50%)
Response time of information system, *n*/*N* (%)	
Slow	41/253 (16.21%)
Moderate	170/253 (67.19%)
Fast	42/253 (16.60%)
Information system crashes, *n*/*N* (%)	
No	29/254 (11.42%)
Yes	225/254 (88.58%)
Ease of use of information system, *n*/*N* (%)	
No	37/256 (14.45%)
Yes	219/256 (85.55%)

BHU: Basic Health Unit, FHU: Family Health Unit, SESAP: State Public Department of Health; *n*: Sample subset; *N*: Full sample; %: Percentage. The full sample of the study is 256, variables different from this indicate invalid data (for example, markings “I do not know” or “Not applicable”).

**Table 3:** Digital health care actions, temporality, target audience, suitability of computer equipment and physical structure, ICT tools used, suitability of information system, existence of protocols, relevance of actions, suitability of tools, and digital health resolvability.

Characteristics	*n*/*N* (%)
Implementation of a digital health project (**structured initiative with defined goals, timeline and allocated resources**) or set of actions (**non-systematic digital practices without formal integration into an institutional plan**) in the health unit by PHC health teams, *n*/*N* (%)	
No	149/205 (72.68%)
Yes	56/205 (27.32%)
Temporality of digital health actions, *n*/*N* (%)	
Before the COVID-19 pandemic	13/57 (22.81%)
During the period of social distancing and temporary closure of health services in the COVID-19 pandemic (from 2020 to the first half of 2022)	38/57 (66.67%)
From the second half of 2022	13/57 (22.81%)
Digital actions (individual and collective) (in addition to the use of e-SUS/PEC) performed by teams in remote care during the critical period of the COVID-19 pandemic (2020—1st half of 2022), *n*/*N* (%)	
Guidance and/or remote care of COVID-19 cases	69/111 (62.16%)
Telecare/teleconsultation for COVID-19	32/111 (28.83%)
Telecare/teleconsultation for non-COVID-19 cases	7/111 (6.31%)
Continuity of treatment and monitoring of chronic non-communicable disease cases	23/111 (20.72%)
Disease prevention actions	33/111 (29.73%)
Health promotion actions	36/111 (32.43%)
Target audience of actions, *n*/*N* (%)	
Children	50/102 (49.02%)
Adolescents	48/102 (47.06%)
Adults	90/102 (88.24%)
Women	66/102 (64.71%)
Men	58/102 (56.86%)
Elderly people	78/102 (76.47%)
Continuity of actions after the critical period of the pandemic (from the 2nd half of 2022), *n*/*N* (%)	
No	72/97 (74.23%)
Yes	25/97 (25.77%)
Digital health actions performed today (in addition to the use of e-SUS/PEC), *n*/*N* (%)	
Guidance and/or remote care of COVID-19 cases	9/68 (13.24%)
Telecare/teleconsultation for non-COVID-19 cases	2/68 (2.94%)
Continuity of treatment and monitoring of chronic non-communicable disease cases	26/68 (38.24%)
Disease prevention actions	48/68 (70.59%)
Health promotion action	52/68 (76.47%)
Target audience	
Children	37/76 (48.68%)
Adolescents	40/76 (52.63%)
Adults	70/76 (92.11%)
Women	61/76 (80.26%)
Men	51/76 (67.11%)
Elderly people	53/76 (69.74%)
Suitability of the health unit’s computer equipment to receive on-site and remote demands, *n*/*N* (%)	
No	134/175 (76.57%)
Yes	41/175 (23.43%)
Suitability of the health unit’s physical infrastructure to receive on-site and remote demands, *n*/*N* (%)	
No	162/195 (83.08%)
Yes	33/195 (16.92%)
Digital ICT tools used in remote care, *n*/*N* (%)	
Telephone calls	66/110 (60.00%)
Videos	26/110 (23.64%)
SMS messages	11/110 (10.00%)
Social media (WhatsApp^®^)	72/110 (65.45%)
Social media (Instagram)	55/110 (50.00%)
Social media (Facebook)	9/110 (8.18%)
Social media (others)	6/110 (5.45%)
Portals	2/110 (1.82%)
Google Meet	20/110 (18.18%)
Cloud computing, “clouds”, that is, internet data processing	9/110 (8.18%)
Suitability of the information system used in on-site and remote services, *n*/*N* (%)	
No	81/129 (62.79%)
Yes	48/129 (37.21%)
Existence of protocols for the organization of remote care actions, *n*/*N* (%)	
No	117/138 (84.78%)
Yes	21/138 (15.22%)
Relevance of care actions in relation to the faced health problems, *n*/*N* (%)	
Sometimes	32/106 (30.19%)
Most of the time	43/106 (40.57%)
Not relevant	9/106 (8.49%)
A few times	14/106 (13.21%)
Always	8/106 (7.55%)
Suitability of the used digital tools, *n*/*N* (%)	
Sometimes	35/119 (29.41%)
Most of the time	52/119 (43.70%)
Not suitable	14/119 (11.76%)
A few times	14/119 (11.76%)
Always	4/119 (3.36%)
Digital health resolvability, *n*/*N* (%)	
No	33/154 (21.43%)
Yes	121/154 (78.57%)

PHC: Primary Health Care; ICTs: Information and Communication Technologies; PEC: Electronic Citizen Record (PEC, as per its Portuguese acronym); *n*: Sample subset; *N*: Full sample; %: Percentage. Full sample of the study is 256, variables different from this indicate invalid data (for example, markings “I do not know” or “Not applicable”).

**Table 4:** Relationship among professionals who perform digital health actions today.

		Performance of Digital Health Actions Today		
Characteristics	Total, *N* = 256	No, *n* = 188	Yes, *n* = 68	*p*-Value
Age group, *n*/*N* (%)				0.155 ^1^
+40	113/256 (44%)	78/188 (41%)	35/68 (51%)	
20–40	143/256 (56%)	110/188 (59%)	33/68 (49%)	
Link, *n*/*N* (%)				0.355 ^1^
Statutory	129/256 (50%)	98/188 (52%)	31/68 (46%)	
Other	127/256 (50%)	90/188 (48%)	37/68 (54%)	
Training, *n*/*N* (%)				0.218 ^1^
Nurse	138/256 (54%)	97/188 (52%)	41/68 (60%)	
Physician	118/256 (46%)	91/188 (48%)	27/68 (40%)	
Graduate training, *n*/*N* (%)				0.165 ^1^
No	65/256 (25%)	52/188 (28%)	13/68 (19%)	
Yes	191/256 (75%)	136/188 (72%)	55/68 (81%)	
Course or training in the use of digital health tools, *n*/*N* (%)				0.205 ^1^
No	202/256 (79%)	152/188 (81%)	50/68 (74%)	
Yes	54/256 (21%)	36/188 (19%)	18/68 (26%)	
Suitability of the BHU computer equipment to receive on-site and remote demands, *n*/*N* (%)				0.013 ^1^
No	134/175 (77%)	102/125 (82%)	32/50 (64%)	
Yes	41/175 (23%)	23/125 (18%)	18/50 (36%)	
Suitability of the BHU physical infrastructure to receive on-site and remote demands, *n*/*N* (%)				<0.001 ^1^
No	162/195 (83%)	122/137 (89%)	40/58 (69%)	
Yes	33/195 (17%)	15/137 (11%)	18/58 (31%)	
Existence of protocols, guidelines or regulations for the organization of remote care actions, *n*/*N* (%)				<0.001 ^1^
No	162/195 (83%)	122/137 (89%)	40/58 (69%)	
Yes	33/195 (17%)	15/137 (11%)	18/58 (31%)	
Digital health resolvability, *n*/*N* (%)				0.645 ^1^
No	33/154 (21%)	19/94 (20%)	14/60 (23%)	
Yes	121/154 (79%)	75/94 (80%)	46/60 (77%)	

BHU: Basic Health Unit; *n*: Sample subset; *N*: Full sample; %: Percentage; ^1^ Fisher’s exact test. Variables with *N* different from the total of the category indicate invalid data (for example, markings “I do not know” or “Not applicable”).

**Table 5:** Relationship among professionals who took a course or training for the use of digital health tools in the context of digital health actions during the COVID-19 pandemic, its continuity, resolvability and statistical significance (*p*).

	Course or Training for the Use of Digital Health Tools		
Characteristics	No *n* = 202	Yes *n* = 54	*p*-Value
Temporality of digital health actions, *n*/*N* (%)			
Before the COVID-19 pandemic	4/36 (11%)	9/21 (43%)	0.009 ^1^
During the period of social distancing and temporary closure of health services in the COVID-19 pandemic (from 2020 to the first half of 2022)	27/36 (75%)	11/21 (52%)	0.081 ^2^
From the second half of 2022	7/36 (19%)	6/21 (29%)	0.518 ^1^
Digital actions (individual and collective) (in addition to the use of PEC) performed by teams in remote care during the critical period of the COVID-19 pandemic (2020—1st half of 2022), *n*/*N* (%)			
Guidance and/or remote care of COVID-19 cases	49/81 (60%)	20/30 (67%)	0.551 ^2^
Telecare/Teleconsultation for COVID-19	24/81 (30%)	8/30 (27%)	0.760 ^2^
Telecare/Teleconsultation for non-COVID-19 cases	2/81 (2.5%)	5/30 (17%)	0.015 ^1^
Continuity of treatment and monitoring of Chronic Non-Communicable Disease cases	19/81 (23%)	4/30 (13%)	0.243 ^2^
Disease prevention actions	26/81 (32%)	7/30 (23%)	0.370 ^2^
Health promotion actions	28/81 (35%)	8/30 (27%)	0.430 ^2^
Digital health actions performed today, *n*/*N* (%)			
Guidance and/or remote care of COVID-19 cases	5/50 (10%)	4/18 (22%)	0.231 ^1^
Telecare/Teleconsultation for non-COVID-19 cases	0/50 (0%)	2/18 (11%)	0.067 ^1^
Continuity of treatment and monitoring of Chronic Non-Communicable Disease cases	17/50 (34%)	9/18 (50%)	0.231 ^2^
Disease prevention actions	39/50 (78%)	9/18 (50%)	0.025 ^2^
Health promotion actions	41/50 (82%)	11/18 (61%)	0.105 ^1^

*n*: Sample subset; *N*: Full sample; % Percentage; ^1^ Fisher’s exact test; ^2^ Chi-square test of independence; Variables with *N* different from the total of the study indicate invalid data (for example, markings “I do not know” or “Not applicable”).

**Table 6:** Nurses and physicians in the context of digital health actions during the COVID-19 pandemic, their continuity and statistical significance (*p*).

	Training		
Characteristics	Nurses *n* = 138	Physicians *n* = 118	*p*-Value
Temporality of digital health actions, *n*/*N* (%)			
Before the COVID-19 pandemic	9/40 (23%)	4/17 (24%)	>0.999 ^1^
During the period of social distancing and temporary closure of health services in the COVID-19 pandemic (from 2020 to the first half of 2022)	29/40 (73%)	9/17 (53%)	0.152 ^2^
From the second half of 2022	8/40 (20%)	5/17 (29%)	0.499 ^1^
Digital actions (individual and collective) (in addition to the use of PEC) performed by teams in remote care during the critical period of the COVID-19 pandemic (2020—1st half of 2022), *n*/*N* (%)			
Guidance and/or remote care of COVID-19 cases	50/74 (68%)	19/37 (51%)	0.097 ^2^
Telecare/teleconsultation for COVID-19	19/74 (26%)	13/37 (35%)	0.300 ^2^
Telecare/teleconsultation for non-COVID-19 cases	6/74 (8,1%)	1/37 (2,7%)	0.421 ^1^
Continuity of treatment and monitoring of chronic non-communicable disease cases	14/74 (19%)	9/37 (24%)	0.508 ^2^
Disease prevention actions	23/74 (31%)	10/37 (27%)	0.660 ^2^
Health promotion actions	23/74 (31%)	13/37 (35%)	0.667 ^2^
Digital health actions per-formed today, *n*/*N* (%)			
Guidance and/or remote care of COVID-19 cases	8/41 (20%)	1/27 (3,7%)	0.076 ^1^
Telecare/teleconsultation for non-COVID-19 cases	1/41 (2,4%)	1/27 (3,7%)	>0.999 ^1^
Continuity of treatment and monitoring of chronic non-communicable disease cases	12/41 (29%)	14/27 (52%)	0.061 ^2^
Disease prevention actions	28/41 (68%)	20/27 (74%)	0.609 ^2^
Health promotion actions	30/41 (73%)	22/27 (81%)	0.429 ^2^
Suitability of the BHU computer equipment to receive on-site and remote demands, *n*/*N* (%)			
No	71/97 (73%)	63/78 (81%)	0.240 ^2^
Yes	26/97 (27%)	15/78 (19%)	
Suitability of the BHU physical infrastructure to receive on-site and remote demands, *n*/*N* (%)			
No	88/112 (79%)	74/83 (89%)	0.051 ^2^
Yes	24/112 (21%)	9/83 (11%)	

*n*: Absolute frequency; *N*: Valid data; %: Percentage. ^1^ Fisher’s exact test. ^2^ Independence Chi-square test. Variables with *N* different from the study total indicate invalid data (for example, markings “I do not know” or “Not applicable”).

## Data Availability

The datasets generated and/or analyzed during the current study are available in the FIGSHARE repository, https://doi.org/10.6084/m9.figshare.28447277.v2. The survey script is available in the supplementary material at the link: https://www.frontiersin.org/journals/public-health/articles/10.3389/fpubh.2024.1304148/full#supplementary-material, accessed on 22 June 2025 (Data Sheet 3, INSTRUMENT 1).

## Abbreviations

PHC	Primary Health Care
REC	Research Ethics Committee
NCDs	Chronic Non-Communicable Diseases
eAP	Primary Care Teams (as per its Portuguese acronym)
DHS	Digital Health Strategy
eSF	Family Health Teams (as per its Portuguese acronym)
HUOL	Onofre Lopes University Hospital (as per its Portuguese acronym)
WHO	World Health Organization
PEC	Electronic Citizen Record (as per its Portuguese acronym)
PNIIS	National Policy on Health Information and Informatics (as per its Portuguese acronym)
RN	State of Rio Grande do Norte
SEIDIGI	Information and Digital Department of Health (as per its Portuguese acronym)
SESAP	State Public Department of Health (as per its Portuguese acronym)
SISAB	Health Information System for Primary Care (as per its Portuguese acronym)
SPSS	Statistical Package for the Social Sciences
SUS	Brazilian Unified Health System (as per its Portuguese acronym)
ICTs	Information and Communication Technologies (ICTs)
UFRN	Federal University of Rio Grande do Norte (as per its Portuguese acronym)
URSAP	Regional Health Units (as per its Portuguese acronym)
FHU	Family Health Units
